# Watermelon Seed in Trachea

**Published:** 1886-08

**Authors:** James Pickett

**Affiliations:** Burleson, Texas


### WATERMELON SEED IN TRACHEA.

BURLESON, TEXAS, July 27, 1886.

EDITOR DANIEL'S MEDICAL JOURNAL.

A few days ago while Mr. H. Sparkman was eating some watermelon, he accidentally sucked a seed and a portion of the pulp into the trachea. Nature at once set up a violent effort to expel the seed and pulp but failed to succeed.

Mr. Sparkman immediately consulted me and I gave him Pulv. Ipecac until I had produced gentle nausea; then I caused some dry Scotch snuff to be blown into his throat. This caused violent coughing, and the seed and pulp were promptly expelled.

Thinking this plan of treatment is a little out of the usual line, I have written the above for publication if you think it worthy of space in your valuable Journal.

Very respectfully,

JAMES PICKETT, M. D.

